# Functional Dynamic Contrast-Enhanced Magnetic Resonance Imaging in an Animal Model of Brain Metastases: A Pilot Study

**DOI:** 10.1371/journal.pone.0109308

**Published:** 2014-10-03

**Authors:** Linfeng Zheng, Pengpeng Sun, Sujuan Zheng, Yuedong Han, Guixiang Zhang

**Affiliations:** 1 Department of Radiology, Shanghai First People’s Hospital, Shanghai Jiao Tong University, Shanghai, China; 2 Department of Radiology, Affiliated Hospital of Binzhou Medical University, Binzhou, Shandong, China; 3 Dengfeng People’s Hospital, Zhengzhou, Henan, China; 4 Department of Radiology, General Hospital of Lanzhou Military Region, Lanzhou, Gansu, China; Northwestern University Feinberg School of Medicine, United States of America

## Abstract

**Background:**

Brain metastasis is a common disease with a poor prognosis. The purpose of this study is to test feasibility and safety of the animal models for brain metastases and to use dynamic contrast-enhanced magnetic resonance imaging (DCE-MRI) to enhance detection of brain metastases.

**Methods:**

With approval from the institutional animal ethics committee, 18 New Zealand rabbits were randomly divided into three groups: Group A received an intra-carotid infusion (ICI) of mannitol followed by VX2 cells; group B received successive ICI of mannitol and heparin followed by VX2 cells; and group C received an ICI of normal saline. The survival rate and clinical symptoms were recorded after inoculation. After two weeks, conventional MRI and DCE-MRI were performed using 3.0 Tesla scanner. The number of tumors and detection rate were analyzed. After MRI measurements, the tumors were stained with hematoxylin-eosin.

**Results:**

No rabbits died during the procedure. The rabbits had common symptoms, including loss of appetite, lassitude and lethargy, etc. at 10.8±1.8 days and 8.4±1.5 days post-inoculation in group A and B, respectively. Each animal in groups A and B re-gained the lost weight within 14 days. Brain metastases could be detected by MRI at 14 days post-inoculation in both groups A and B, with metastases manifesting as nodules in the brain parenchyma and thickening in the meninges. DCE-MRI increased the total detection of tumors compared to non-contrast MRI (P<0.05). The detection rates of T1-weighted image, T2-weighted image and DCE-MRI were 12%, 32% and 100%, respectively (P<0.05). Necropsy revealed nodules or thickening meninges in the gross samples and VX2 tumor cytomorphologic features in the slides, which were consistent with the MRI results.

**Conclusions:**

The VX2 rabbit model of brain metastases is feasible, as verified by MRI and pathologic findings, and may be a suitable platform for future studies of brain metastases. Functional DCE-MRI can be used to evaluate brain metastases in a rabbit model.

## Introduction

Brain metastases are secondary brain tumors that result from the spread of malignant tumors from locations other than the brain, and they are more common than primary brain tumors [1]–[4]. According to statistics, 20–40 percent of malignant tumor cases will undergo brain metastases over the course of the diseases [3], [4]. Of these cases, only approximately 2/3 cases have symptoms, such as headache, seizures, nausea and vomiting, etc., or focal neurological signs that lead to the diagnosis. However, the optimal management strategy for these patients cannot be determined at the stage of their diagnosis [1], [5]. Therefore, there is a clinical need for the development of effective modalities that can provide a reliable diagnosis of brain metastases at an early stage.

Of the available diagnostic methods for brain metastases, imaging modalities play an important role in non-invasive diagnosis and treatment management [1], [3], [6]–[9]. Computed tomography (CT) has been widely used in the diagnosis, staging, monitoring, therapeutic effect evaluation and follow-up due to its accessibility and affordability [6], [7]. Routine cranial CT and contrast-enhanced CT (CECT) can delineate lesions for larger tumor metastases. However, for small metastases or those located in special locations, such as the posterior fossa, CT cannot achieve ideal delineation. Studies have shown that both routine CT and CECT have a limited sensitivity, which is approximately 80% [6]. Positron emission tomography (PET) and its hybrid integrated image techniques, including PET/CT and PET/magnetic resonance imaging (MRI), are now important imaging modalities for diagnosis and staging as well as for providing prognostic information based on the response to treatment [9]–[11]. However, with respect to its application in brain metastases, PET is not an ideal tool for detecting brain metastases due to the high uptake of fluorodeoxyglucose (FDG) by unaffected brain tissue. Numerous reports have shown that the sensitivity was approximately 60–75%, and the specificity was approximately 83% for the identification of cerebral metastases [8], [11]. Rohren et al.’s study reported that only 61% of metastatic lesions in the brain were identified with PET, and it was difficult to detect small lesions [12]. Therefore, it is necessary to further investigate other non-invasive imaging modalities to improve brain metastases management.

With the development and improvement of MRI scanners and sequences, MRI has achieved substantial spatial resolution and extraordinary gray-white matter contrast capacity, among other features [5], [7], [8], [13]. At present, MRI is widely used as a non-invasive method for detecting brain metastases. MRI is more sensitive and can offer more anatomical and functional information for evaluating the presence of brain metastases and their response to treatment compared to CT and PET [1]–[3], [5], [8], [14]. Although clinicians and radiologists have reached a consensus that MRI is a useful and effective tool for evaluating brain metastases, there is increasing controversy about the role of non-contrast MRI and contrast-enhanced MRI in detecting brain metastases, especially with the use of functional dynamic contrast-enhanced MRI (DCE-MRI) [15]. Up to now, no standard criteria have identified DCE-MRI as the preferred method for assessing brain metastases. There is a clinical need for validation of DCE-MRI that can serve as a preferred method to evaluate brain metastases in an animal model. Comparing with murine model for brain metastases, the rabbit model has the following advantages, including (a) being docile and non-aggressive and hence convenient to handle and observe behavior; (b) being relatively inexpensive to purchase, house, and maintain; (c) having less health problems; and (d) being easy for transplantation or transmission of tumors [16]–[22]. Therefore, the purpose of this study is to test feasibility and safety of the animal models for brain metastases and to use DCE-MRI to enhance detection of brain metastases in a rabbit animal brain metastases model.

## Materials and Methods

### Animals

This study was carried out in strict accordance with the recommendations in the guide for the care and use of laboratory animals by the authority of the People’s Republic of China. With approval from the animal ethics committee at First People’s Hospital, Shanghai Jiao Tong University for the protocol (Permit Number: Radiol-1-2009), 18 healthy adult New Zealand white rabbits (female, weighing 2.0–2.3 kg; purchased from Shanghai Baomu Experimental Animal Farm, Shanghai, China) were used in our experiments and then randomly divided into three groups (groups A, B and C; each group n = 6), which received different treatments. All surgery was performed under No. 2 Sumianxin and ketamine (1∶2 v/v; 0.4 ml/kg) anesthesia.

### VX2 tumor cell suspensions processing

VX2 tumors were initially grown in the right hind limb of two donor rabbits (maintained at our institute). The donor rabbit with a VX2 tumor that was approximately 1–2 cm diameter was anesthetized with No. 2 Sumianxin and ketamine (1∶2 v/v; 0.4 ml/kg) and fixed on the operation table. After shaving and disinfection of the right hind limb, the tumor was exposed and we cut the tumor capsule. Parts of the tumor tissue from the tumor margin were removed and washed twice in a sterile container with normal saline (NS) to deplete blood. Then, the tumor tissue was cut into small pieces with scissors and the minced tumor was pulverized using the back end of the syringe plunger, in a sterile container, and 1 ml NS was added to the container. After mixing and filtering with 200 mesh sterile stainless steel screen, tumor suspensions was prepared. The cell numbers of the tumor suspensions were counted using a hemocytometer and adjusted to 10^6^–10^8^ cells/ml with NS for the following study [23].

### Establishment of the animal model

**Figure 1:**
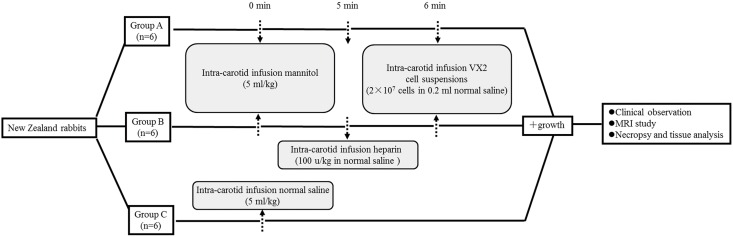
Flow chart of the experiment design.

The rabbits in three different groups were anesthetized as noted above. After anesthesia, the side and anterior regions of the neck were shaved on each rabbit which was placed in the supine position on the operation table. Lateral cervical access was achieved, extending approximately 3 cm, and we carefully dissected the tissue. The left carotid artery was exposed, and two suture lines were placed under the carotid artery. An I.V. catheter (Tyco healthcare, Mansfield, MA, USA) was placed in the left carotid artery and fixed with one of two suture lines that approached the cranial direction. After gently withdrawing the preloaded stylet from the catheter, the rabbits were given different treatments as follows: Group A: first received an intra-carotid infusion (ICI) of mannitol (5 ml/kg) via catheter; then, after 5 min, received an ICI of the VX2 cell suspensions (2×10^7^ cells in 0.2 ml of NS); Group B: first received an ICI mannitol (5 ml/kg) via catheter; then, after 5 min, received an ICI of heparin (100 u/kg in saline) prior to receiving an ICI of the VX2 cell suspensions (2×10^7^ cells in 0.2 ml of NS); Group C: only received an ICI of NS (5 ml/kg) (Fig. 1). Then, we removed the catheter from the artery and promptly ligated the artery. The cervical incision was closed with simple con­tinuous sutures for the muscle and fascia layer and simple interrupted suturing of the skin layer. After operation, each rabbit was subcutaneously injected with ketoprofen (1.0 mg/kg, once daily) for three days, and all efforts were made to minimize suffering. The experimental rabbits were returned to storage facilities for clinical observation (one time a day) and examined for the next 30 days (Fig. 1).

### MRI

The MRI examination started 2–3 weeks after intra-arterial inoculation. All rabbits’ MR studies were carried out with a 3.0 Tesla clinical MRI scanner (GE Medical Systems, Milwaukee, WI, USA). The rabbits were anesthetized with a mixture of No. 2 Sumianxin and ketamine (0.4 ml/kg) and were placed in the supine position in an eight-channel knee coil. The non-contrast T1-weighted (T1W) and T2-weighted (T2W) sequences were obtained with the following parameters. For non-contrast T1W sequence, T1 Fast fluid attenuated inversion recovery (FLAIR) sequence was obtained with field of view (FOV) 14 cm×10.5 cm, matrix 512×512, repetition time (TR)/echo time (TE) 1818/8.7 ms, inversion time (TI) 1000 ms, section thickness 2.0 mm, interval 0.5 mm, slice 30, and number of excitations (NEX) 2. T2W sequence was obtained with fast spin-echo sequence, FOV 14 cm×10.5 cm, matrix 320×288, TR/TE 4440/76.2 ms, section thickness 2.0 mm, interval 0.5 mm, slice 30, and NEX 2. Before DCE-MRI scanning, the pre-contrast T1 mapping images were acquired with fast low angle shot (FLASH) sequence. After the pre-contrast T1-mapping scanning, the DCE-MRI scan was then performed by dynamic acquisition with T1W sequence using gadolinium-diethylenetriamine pentaacetic acid (Gd-DTPA; Magnevist, 0.1 mmol/kg, Bayer Schering Pharma AG, Berlin, Germany). Intravenous bolus of contrast agent was administered by hand at a 2 ml/min flow rate under the same positions as used in the pre-contrast T1-mapping protocol [24]. The MR image features, including the location, size, and signal intensity of the brain metastases, were analyzed by two experienced radiologists (LZ and GZ, with 5 and 20 years of MRI experience, respectively) using GE FUNCTOOL 4.4.05 software (GE Medical Systems, Milwaukee, WI, USA). The brain metastases were recorded as brain and meningeal metastases according to the location and features of brain tumors.

### Necropsy and pathological study

At the end of the final MRI examination, the rabbits were deeply anesthetized and euthanized with sodium pentobarbital (120 mg/kg, intravenous injection). All rabbits were perfused intracardially with NS (approximate 1000 ml) followed by 5000 ml of 10% neutral formalin. The rabbit brains were dissected and immersed in 10% hydrochloric acid solution for one week of decalcification. After gross observation with a thick brain section, the samples were dehydrated by gradient alcohol and embedded in paraffin. Four micron thick slices were cut and standard hematoxylin-eosin (HE) staining was performed. Finally, the slices were observed under light microscopy and digital pictures were captured (LZ and one pathologist).

### Statistical analysis

The statistical significance of the difference was tested using the student *t* test, Chi-square test or non-parametric test according to the different data by using SPSS 19.0 software (SPSS Inc., Chicago, IL, USA). P<0.05 was considered statistically significant.

## Results

### Survival rate and clinical symptoms after inoculation

Of 18 experimental rabbits, no rabbits died during the observation time after the model procedure. In groups A and B, all but one rabbit in group A displayed loss of appetite, lassitude, lethargy and reduction of movement after the intra-arterial delivery of the VX2 cell suspensions at 10.8±1.8 days post-inoculation (group A) and 8.4±1.5 days post-inoculation (group B); each rabbit re-gained the lost weight by 14 days post-inoculation. One rabbit in group A and one rabbit in group B developed swelling in the maxillofacial region by 14 days post-inoculation. One rabbit in group B developed eye symptoms, including tears, exophthalmos and conjunctival congestion, which accompany swelling in the maxillofacial region by 14 days post-inoculation (Table 1). No clinical evidence of illness was observed in the control group (group C) rabbits (Table 1).

**Table 1:** Clinical symptoms and occurrence days for each group of rabbits after the model procedure.

Group		Clinical symptoms							Total
		Loss ofappetite	Lassitude	Lethargy	Reduction ofmovement	Lostweight	Swelling inmaxillofacial region	Eyesymptoms	
Group A	Number (n/Total)	5/6				6/6	1/6	0/6	6
	Occurrence days	10.8±1.8				14	14	N/A^a^	
Group B	Number (n/Total)	6/6				6/6	1/6	1/6	6
	Occurrence days	8.4±1.5				14	14	14	
Group C	Number (n/Total)	0/6							6
	Occurrence days	N/A^a^							

a N/A = not applicable.

### Brain metastases features and MRI results

**Figure 2:**
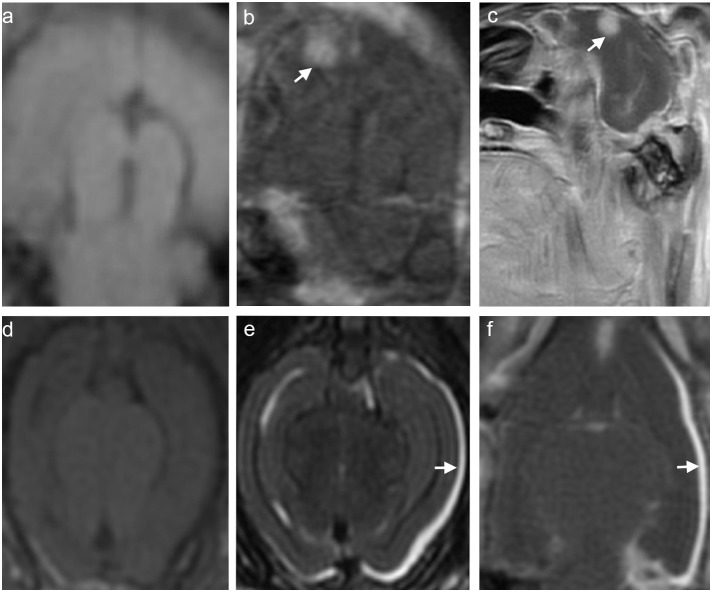
Representative magnetic resonance (MR) images of a brain parenchymal metastatic tumor (a–c) and meningeal metastases (d–f). (a) Non-contrast coronal T1-weighted (T1W) image. (b) Contrast enhanced coronal T1W image. (c) Contrast enhanced sagittal T1W image. (d) Non-contrast axial T1W image. (e) Non-contrast axial T2-weighted image. (f) Contrast enhanced coronal T1W image. The white arrow in (b, c) and (e, f) indicate the tumor sites.

Brain metastases were observed by MRI in both groups A and B two weeks after inoculation. No tumors were found in group C. The tumors manifested as nodules in the brain parenchyma (Fig. 2a–c) and line-like or nodule thickening in the meninges (Fig. 2d–f). In group A, the average detectable numbers of brain parenchymal tumor nodules were 2 and 3, while thickening of the meninges were 1 and 4 by non-contrast MRI and DCE-MRI, respectively (Table 2). In group B, the average detectable numbers of brain parenchymal tumor nodules were 6 and 22, while thickening of the meninges were 2 and 4 by non-contrast MRI and DCE-MRI, respectively (Table 2). There was a statistically significant difference in the brain parenchymal tumor nodules between groups A and B (Table 2, P<0.05). The diameter of the brain parenchymal tumor nodules ranged from 1.5 to 7.0 mm in group A (4.0±0.5 mm, mean±standard error of the mean [SEM]) and from 1.5 to 5.0 mm in group B (3.7±0.4 mm). There was no significant differences in the diameters of the brain parenchymal nodules between groups A and B (P>0.05).

**Table 2:** The average number of detectable tumors for different types of brain metastases in group A and B by non-contrast magnetic resonance imaging (MRI) and dynamic contrast-enhanced MRI (DCE-MRI).

Group	Non-contrast MRI			DCE-MRI	
	Brain parenchymal nodule		Thickening meninges	Brain parenchymal nodule	Thickening meninges
Group A	2	1		3	4
Group B	6^a^	2		22^a^	4

a P<0.05 compared with brain parenchymal nodules in group A.

**Figure 3:**
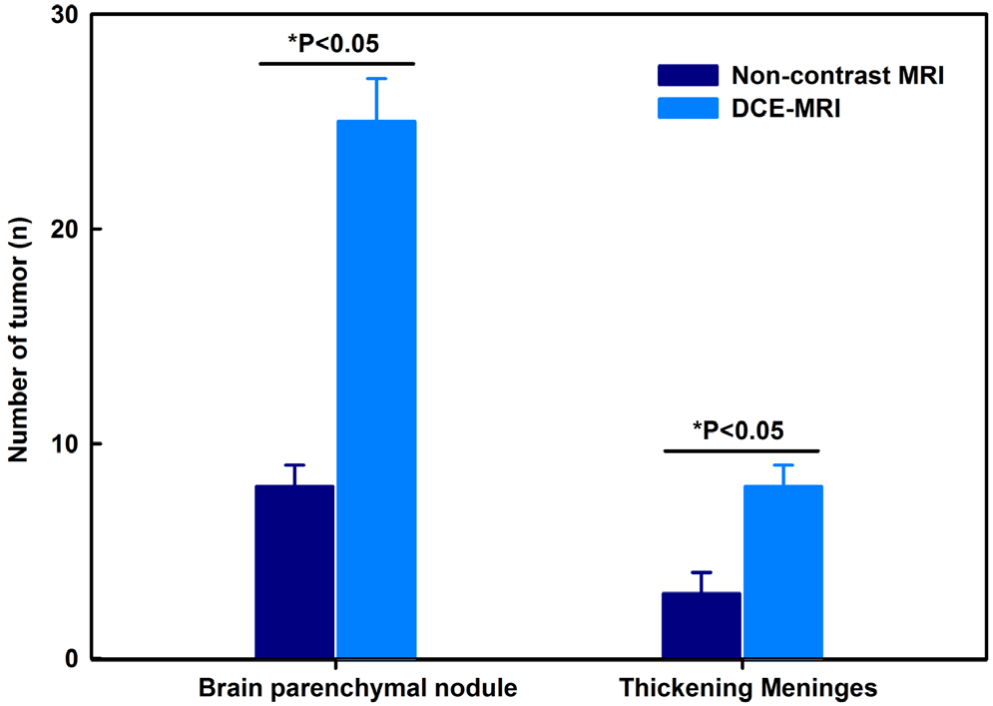
The number of brain parenchymal metastatic tumor nodules and thickening meninges by non-contrast magnetic resonance imaging (MRI) and dynamic contrast-enhanced MRI (DCE-MRI).

The detectable minimal diameter of tumor was 1.5 mm by DCE-MRI and was 3 mm by non-contrast MRI. For brain parenchymal metastatic nodules, the detected numbers of tumors by non-contrast MRI and DCE-MRI were 8±1 and 25±2, respectively. For thickening meninges of brain metastases, the tumor numbers by non-contrast MRI and DCE-MRI were 3±1 and 8±1, respectively. DCE-MRI obviously increased the total numbers of tumor detection compared to non-contrast MRI (Fig. 3). For both the number of brain parenchymal nodules and thickening of the meninges for brain metastases, the differences between non-contrast MRI and DCE-MRI were statistically significant (Fig. 3, P<0.05). To compare the detection rate in different sequences, the detection rates of T1W image, T2W image and DCE-MRI were 12%, 32% and 100%, respectively (P<0.05).

### Necropsy and pathological findings

**Figure 4:**
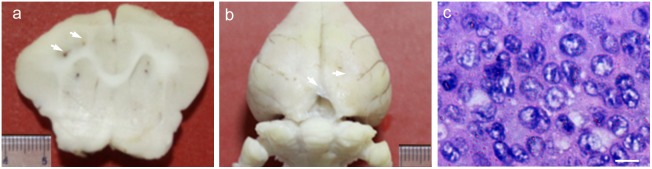
Gross sample (a, b) and hematoxylin-eosin (HE) staining (c) of brain metastases. The white arrows in (a, b) indicate the nodule in brain parenchyma (a) and the thickening meninges (b), respectively. The scale bar in (c) = 20 µm.

The gross brain specimen of the rabbits showed tumor nodules in the thick section of brain (Fig. 4a) and thickening meninges (Fig. 4b), which was consistent with the MRI results. HE staining of these samples showed typical VX2 tumor cytomorphology, including different nuclear sizes, increasing ratio of the nucleus to cytoplasm and atypia (Fig. 4c).

## Discussion

In this study, we have shown that functional DCE-MRI can be used as a modality to detect brain metastases in a rabbit model. We successfully established the feasibility and safety of VX2 rabbit model of brain metastases by intra-arterial infusion of tumor cells. We have also shown that functional DCE-MRI can increase the detection rate for both brain parenchymal metastases and meningeal metastases in an animal brain metastases model.

Animal models are crucial for improving the diagnosis and treatment of brain metastases. In our team’s and Grossman team’s previous studies, brain metastases were established in an animal model via cerebrospinal fluid (CSF) injection of tumor cells in rabbits. But the higher mortality and diffuse metastases via CSF to the spinal cord limited its broad application [25]–[27]. In a recent mechanism study of brain metastases, the authors reported that the tumor cell survival in the bloodstream is the key step of the complicated “metastatic cascade” [28],[29]. In this present study, we tried to establish a brain metastases model via the injection of VX2 tumor cell suspensions via the carotid artery. We directly injected tumor cells into carotid arteries, which can mimic the brain metastatic process as well as avoid the disadvantages of CSF inoculation. Breast cancer brain metastasis in BALB/c mice and Lewis lung carcinoma brain metastasis in C57BL/6Ncrj mice with internal carotid artery injection was also reported by Lorger et al. [30] and Saito et al. [31], respectively. This intra-arterial injection procedure via carotid artery avoids dissemination in the lung, which would otherwise happen when other vessels are used to establish the animal model. This is a relatively superior approach for brain metastases models. There was no obvious animal death during our study period and this inoculation procedure minimized the spread of tumor to sites other than the brain. Therefore, the results presented here indicates that the VX2 rabbit model of brain metastases is feasible, as verified with MRI and pathologic findings, which may be a suitable platform for future studies of brain metastases.

The blood-brain barrier (BBB) is a key barrier to prevent the hematogenous dissemination of tumor cells into the brain. During the formation of brain metastases, tumor cells need to penetrate the BBB and then invade and grow in the brain microenvironment [28], [29]. Therefore, we used mannitol, a diuretic, to open the BBB and facilitate tumor cell movement across the BBB and into the brain [32]. The results of this study showed that this method could be useful for establishing brain metastases in rabbits. Additionally, we also found that the number of tumor metastases in the meninges was not different between groups A and B. However, the number of brain parenchymal tumor nodules in group B was higher than in group A. Because the only difference was the treatment with heparin in the procedure for group B, we speculated that heparin maybe play a role in preventing the tumor cell coagulation in the blood and hence improving the penetration of tumor cells into the brain. Nevertheless, the specific mechanism of heparin function merits further study [33].

In the present study, we report that functional DCE-MRI obviously increases the detectable numbers of brain tumor nodules, and decreases the minimal diameter of tumor nodules to 1.5 mm. These findings are consistent with previously published studies [5], [7]. According to the guidelines for brain metastases, the number of tumors is one of key indexes for planning therapy and evaluating prognosis in addition to age, tumor size and site, the status of primary tumor and Karnofsky performance status (KPS), etc. [3], [5], [34]. Studies have shown that approximately 20% of patients with a solitary metastatic brain lesion by other image modalities were re-diagnosed with multiple brain lesions on MRI [35], [36]. Despite the superiority of MRI for the detection of brain metastases, there are some concerns, such as the MRI methods, sequences and sensitivity, etc. that require clarification. Functional DCE-MRI provides better soft-tissue contrast and stronger tumor enhancement when using paramagnetic contrast agents such as gadolinium, resulting in the detection of smaller lesions than conventional MRI [22], [36]. The passive contrast enhancement of tumors by functional DCE-MRI relies on breakdown of the blood-brain barrier (BBB), and most studies have reported that the minimal diameter of clinical detection for brain metastases is 2–5 mm when the BBB is permeable [5], [7]. Our finding of minimal diameter of 1.5 mm for tumor detection approaches this published range. Recently, Sibson’s team reported that metastases with a diameter range of 300–650 µm should be detectable with common clinical 3.0 Tesla MRI [5]. Additionally, some studies have shown that functional DCE-MRI can be used to monitor tumor angiogenesis as well as predicts the therapeutic response and more for brain metastases [22], [37], [38]. Our study supports the notion that functional DCE-MRI can be used to detect smaller brain metastases and to assess tumor response to treatment.

There are several limitations in this study. First, we did not calculate the permeability pharmacokinetic parameters, such as K^trans^, Kep and Ve on DCE-MRI, or assess the tumor relative cerebral blood volume using perfusion-weighted imaging in this pilot study [22]. Therefore, the advantages of using these parameters as a non-invasive imaging biomarker to increase detection of brain metastases were not clearly ascertained. Secondly, we did not compare DCE-MRI with conventional MRI with intravenous (IV) contrast. In this study, the imaging results were compared between DCE-MRI versus non-contrast MRI sequences. The superiority of DCE-MRI in brain tumor detection would have been further supported if DCE-MRI were compared to conventional MRI with IV contrast rather than to non-contrast MRI as reported in the present study. Thirdly, DCE-MRI depends on the gadolinium leakage across the BBB, which is not specific to brain metastases. As an extracellular contrast agent, gadolinium provides less information at the cellular level. The development of a newer contrast agent such as novel iron-based particles may provide more information than traditional gadolinium-based contrast agents. A study by Blasiak et al. [19] showed that targeted iron oxide contrast agents could improve the determination of glioma extent by delineating the tumor rim in a mouse model. Using vascular cell adhesion molecule-1 (VCAM-1) targeted iron oxide molecular probes, this molecular MRI has enabled the early and sensitive detection of brain metastases in murine models of brain metastases [5].

In summary, functional DCE-MRI improves visualization and early detection of brain metastases in a live rabbit model. This study shows a promising non-invasive and translational methodology for diagnosis and treatment of brain metastases.
